# Limited Access to Iodized Salt among the Poor and Disadvantaged in North 24 Parganas District of West Bengal, India

**DOI:** 10.3329/jhpn.v28i4.6043

**Published:** 2010-08

**Authors:** Tapas Kumar Sen, Dilip Kumar Das, Akhil Bandhu Biswas, Indranil Chakrabarty, Sujishnu Mukhopadhyay, Rabindranath Roy

**Affiliations:** ^1^ Department of Health and Family Welfare, Government of West Bengal, India; ^*^ (Deceased); ^2^ R.G. Kar Medical College, Kolkata, India; ^3^ B.S. Medical College, Bankura, West Bengal, India; ^4^ Medical College, Kolkata, West Bengal, India; ^5^ Burdwan Medical College, West Bengal, India

**Keywords:** Community-based studies, Cross-sectional studies, Descriptive studies, Goitre, Iodine, Iodine deficiency, Iodized salt, Socioeconomic factors, India

## Abstract

Iodine deficiency is endemic in West Bengal as evident from earlier studies. This community-based, cross-sectional descriptive study was conducted in North 24 Parganas district during August-November 2005 to assess the consumption of adequately-iodized salt and to ascertain the various factors that influence access to iodized salt. In total, 506 households selected using the multi-stage cluster-sampling technique and all 79 retail shops from where the study households buy salt were surveyed. The iodine content of salt was tested by spot iodine-testing kits. Seventy-three percent of the households consumed salt with adequate iodine content (≥15 ppm). Consumption of adequately-iodized salt was lower among rural residents [prevalence ratio (PR): 0.8, 95% confidence interval (CI) 0.7-0.9], Muslims (PR: 0.8, 95% CI 0.7-0.9), and households with monthly per-capita income of ≤US$ 10 (PR: 0.7, 95% CI 0.6-0.8). Those who heard and were aware of the risk of iodine-deficiency disorders and of the benefit of iodized salt were more likely to use appropriate salt (PR: 1.2, 95% CI 1.1-1.3). Those who were aware of the ban on non-iodized salt were more likely to consume adequately-iodized salt (PR: 1.1, 95% CI 1.01-1.3). The iodine content was higher in salt sold in sealed packets (PR: 2.9, 95% CI 1.8-4.8) and stored on shelves (PR: 1.6, 95% CI 1.3-2.0). Seventy-two percent of the salt samples from the retail shops had the iodine content of ≥15 ppm. The findings indicate that elimination of iodine deficiency will require targeting the vulnerable and poor population.

## INTRODUCTION

Iodine-deficiency disorders (IDDs) are a global problem, affecting the people of 130 of 191 member countries of the World Health Organization (1). It is one of the most common preventable causes of mental retardation globally (2). The most visible manifestation of IDD—goitre—is seen in 13% of the world's population. An estimated 167 million people in India are at risk of IDDs. Of these, 54 million suffer from goitre, two million suffer from cretinism, and 6.6 million children have neurological deficits (3). In a recent study, North 24 Parganas district in West Bengal, located in eastern part of India, was found to be iodine-deficient (4). The district has a mix of urban and rural population and also of different religions. It is a flood-prone area and located at the Gangetic basin bordering the Bay of Bengal (5).

Universal iodization of salt, the mainstay of the intervention, was made compulsory in India in 1998, although it was revoked in 2000 and again reinstated in 2005 (6). Despite those efforts, a study estimated that the coverage of iodized salt in the district was 70% (4). Various studies in some districts of the state of West Bengal also revealed inadequate accessibility to iodized salt (7–12).

The reasons for such inadequate accessibility might be many and diverse, both at the level of consumers and sellers. Various societal, economic and demographic factors may have differential influences on this practice (13). Identification of such factors that influence the accessibility to iodized salt is, thus, essential as it would allow us to formulate a better prevention policy and intervention measures.

With this background, the study was carried out to: estimate the consumption of adequately-iodized salt at the household level in North 24 Parganas district; find out the socioeconomic factors that influence the access to iodized salt at the household level; ascertain any relationship between awareness about iodized salt and IDDs at the household level; and assess the perceptions of retail shop owners about iodized salt and IDDs.

## MATERIALS AND METHODS

This community-based, cross-sectional descriptive study was carried out during August-November 2005 in North 24 Parganas district, West Bengal, India. The study sample comprised selected households from the entire district and all the retail shops from where they buy salts.

We calculated the sample size of the proposed study using the Right Size software (version 2.0.7.2) (14), assuming the consumption of iodized salt at 70% as has been found in a recent study in the same area (4), confidence interval ±5%, confidence coefficient 95%, rate of homogeneity 0.02 (15), and cluster size 20. The total sample size was calculated to be 460 from 23 clusters with an anticipated design effect of 1.38. Considering 10% non-response, the sample size was 506 households. Distributing the households among 23 clusters in villages or wards (defined geographical area in urban locality) meant recruiting 22 households from each cluster. The final sample size of 506 households was selected following the multi-stage cluster-sampling technique. We selected 23 clusters, villages, or wards using the probability proportional to size technique (1), and from each selected cluster, 22 households were identified by simple random sampling from a sampling frame developed from the existing electoral rolls. All the selected areas (villages or wards) were visited before the survey to appraise the appropriate authority of the study and to obtain permission from them.

We designed schedules for collecting relevant data from the households and retail shops and translated both the schedules in local language (Bangla) to help the field workers in data collection. The local language versions were translated back to English for validation. We pretested the schedules and used those to interview one responsible respondent from each household and all the shop-owners and recorded relevant data. The field workers working in the health system of the identified areas were trained on the standard technique of testing iodine in salt and administration of schedules at the household and retail shop levels. The iodine content of the salt samples in parts per million (ppm) was estimated using spot-testing kit (1). For cross-checking, we intended to collect 15% of the salt samples from the households and retail outlets in auto-sealed polythene pouches and estimate the iodine content by titrimetric method (16,17). The test results of titrimetric method were compared with those of rapid test kit. Kappa test was performed to measure the agreement. The field investigators monitored and supervised the entire process of interviewing and testing of salt.

Data were entered into a dBase® file, and data entry was double-checked for errors. Data were analyzed using the Epi Info epidemiological software (version 6.04d) (18). Univariate analysis was performed to examine any statistical significance between the outcome and the response variables. We calculated the prevalence ratios (PRs) with 95% confidence interval for that purpose.

## RESULTS

### Socioeconomic and demographic characteristics

Of the 506 households surveyed for the study, 52.2% were urban and 47.8% were rural households. Of these, 76.5% belonged to Hindus and 23.5% to Muslims, both of which closely resembled the usual distribution pattern in the district in terms of residence and religion. Overall, 24.5% of the Hindu families were of scheduled castes, scheduled tribes, or other backward castes. More than half (53%) of the households had per-capita monthly income of ≤US$ 10.

### Access to iodized salt at the household level

Estimation of the iodine content of the salt samples using spot iodine-testing kit revealed that 369 (72.9%) households were consuming adequately-iodized salt, and the remaining 137 (27.1%) households were consuming either inadequately-iodized or non-iodized salt (Table 1).

**Table 1.** Iodine content of salt at the household and retail shop levels, North 24 Parganas, West Bengal, India, 2005

Iodine content	Households (n=506)		Retail shops (n=152)	
	No.	%	No.	%
Nil	18	3.6	5	3.3
<15 ppm	119	23.5	38	25
≥15 ppm	369	72.9	109	71.7

Further analysis (Table 2) revealed that the consumption of adequately-iodized salt was significantly more by the urban (215/264, 81.4%) population than the rural (154/242, 63.6%) population, by Hindus (295/387, 76.2%) than by Muslims (73/118, 61.9%), by Hindu higher castes (230/292, 78.8%) than by backward castes (65/95, 68.4%), and by families with monthly per-capita income of more than US$ 10 (208/238, 87.4%) than those with lesser income (161/268, 60.1%).

**Table 2.** Socioeconomic factors associated with access to iodized salts, North 24 Parganas, West Bengal, India, 2005

Factor/characteristics	Iodine status of salt				Prevalence ratio (95% CI)
	≥15 ppm		Nil/<15 ppm		
	No.	%	No.	%	
Residence					
Rural (n=242)	154	63.6	88	36.4	0.8 (0.7-0.9)
Urban (n=264)	215	81.4	49	18.6
Religion					
Muslim (n=118)	73	61.9	45	38.1	0.8 (0.7-0.9)
Hindu (n=387)	295	76.2	92	23.8
Caste					
Backward caste* (n=95)	65	68.4	30	31.6	0.9 (0.7-1.0)
General (n=292)	230	78.8	62	21.2
Per-capita monthly income (US$)					
≤10 (n=268)	161	60.1	107	39.9	0.7 (0.6-0.8)
>10 (n=238)	208	87.4	30	12.6

*Backward caste means SC (scheduled caste)/ST (scheduled tribe)/OBC (other backward castes);

CI=Confidence interval;

PPM=Parts per million

The households whose respondents heard about IDDs were used to consume a significantly higher proportion of adequately-iodized salt compared to those who did not (81.3% vs 68.7%; PR: 1.2, 95% CI 1.1-1.3). Similarly, the consumption of iodized salt was higher among those who were aware of any IDD (PR: 1.2, 95% CI 1.1-1.3) and iodized salt (PR: 1.2, 95% CI 1.1-1.4) and its benefits (PR: 1.2, 95% CI 1.1-1.3). The households (83.3%) purchasing loose salt were used to consume inadequately-iodized salt compared to others (22.4%) who always purchased salt in the sealed packet (PR: 2.9, 95% CI 1.8-4.8). Greater proportions of the households with the practice of keeping salt on the shelf (PR: 1.6, 95% CI 1.3-2.0) and in covered pots were consuming adequately-iodized salt. Families whose respondents knew about the ban on selling non-iodized salt were consuming adequately-iodized salt in a higher proportion (PR: 1.1, 95% CI 1.01-1.3) than who did not know (Table 3).

**Table 3.** Knowledge and storage practices of households associated with access to iodized salt, North 24 Parganas, West Bengal, India, 2005

Perception/practice	Iodine status of salts				Prevalence ratio (95% CI)
	≥15 ppm		Nil/<15 ppm		
	No.	%	No.	%	
Heard about IDDs					
Yes (n=171)	139	81.3	32	18.7	
No (n=335)	230	68.7	105	31.3	1.2 (1.1-1.3)
Aware of any IDD					
Aware (n=102)	87	85.3	15	14.7	1.2 (1.1-1.3)
Not aware (n=404)	282	69.8	122	30.2
Heard about iodized salt					
Yes (n=200)	164	82	36	18	1.2 (1.1-1.4)
No (n=306)	205	67	101	33
Any benefit of iodized salt					
Aware (n=77)	66	85.7	11	14.3	1.2 (1.1-1.3)
Not aware (n=429)	303	70.6	126	29.4
Nature of purchase					
Sealed packet (n=460)	357	77.6	103	22.4	2.9 (1.8-4.8)
Loose/both (n=46)	12	26.1	34	73.9
Place of storage					
Shelf (kitchen/store) (n=407)	88	21.6	319	78.4	1.6 (1.3-2.0)
Floor (kitchen/store) (n=89)	45	50.6	44	49.4
Type of container					
Covered pot (n=469)	347	74	122	26	1.2 (0.9-1.6)
Uncovered pot (n=37)	22	59.5	15	40.5
Ban on non-iodized salt					
Aware (n=94)	76	81.9	18	19.1	1.1 (1.01-1.3)
Not aware (n=412)	293	71.1	119	28.9

CI=Confidence interval;

IDDs=Iodine-deficiency disorders

### Access to iodized salt at retail shops

We studied all the 79 retail shops in the selected clusters (villages and wards) from where the study households buy salts. Of these retail shops, 46 (58.2%) were located in the urban areas and 33 (41.8%) in the rural areas. In total, 152 salt samples available on the days of the survey at those 79 shops were tested for iodine content using spot iodine-testing kit.

It was found that 30.4% of the shops were used to selling iodized salt exclusively, 20.3% used to selling both iodized and non-iodized salts, and 48.1% of the shop owners were unsure about the iodine status of salt they sell. About 86.1% of the shop-owners had practice of keeping salt uncovered, and half of them were used to keeping it on the floor. They also disclosed that 20.3% of them still mostly sell loose salt, which has a greater chance of being inadequately iodized. The shop-owners who were knowingly selling non-iodized salt cited the reason of higher price of iodized salt which compelled them to sell the non-iodized brand.

Analysis further revealed that 38% of the shop-owners had knowledge of any IDD, and of them, 53.3% could specify the nature of IDDs. Television (66.7%) was the principal source of information. About 60% of the interviewees actually knew about iodized salt, although 63.4% of them either did not know the specific benefits or knew them wrongly. Even then, 58.2% opined that iodized salt should be consumed.

Surprisingly, of the 79 shops, 87.3% were not aware of the ban on production and sale of non-iodized salt as declared by the Government of India.

Of the 152 salt samples collected from the shops, 109 (71.7%) had the iodine content of ≥15 ppm, 25% had iodine of <15 ppm, and 3.3% had no iodine (Table 1). Of these samples, 26 were loose salts, and only four (15.4%) had the iodine content of ≥15 ppm.

The percentage of agreement regarding estimated iodine content of 114 (17%) salt samples between titrimetric method and rapid test kit was 74.9%, which vouchs for the validity of the method of estimation of iodine in the field condition.

## DISCUSSION

This in-depth community-based study revealed a similar result as was obtained in other studies (7–12) but the overall level of iodization of salt at the consumer level was far from the target of 90% in West Bengal.

The iodine concentration of salt in the rural households was lower than that in the urban households. The similar differences were reported by other studies in India (9,12,19) and in other countries (20,21). It was even lower among those who were unaware of its significance and among the socially- and economically-disadvantaged population. Other reports stated that the consumption of iodized salt varied in respect of socioeconomic condition (13,19)*.* Studies in Bangladesh reported that the economic status of households was related to the consumption of iodized salt (22). Thus, the vulnerable groups are still being exposed to under- or non-iodized salt.

Besides the rural-urban difference and the difference by religion and caste, other factors at the household level which resulted in unequal access to iodized salt were lack of awareness about IDDs, iodized salt and its benefit, purchase of loose salt, and improper practice of storage. Studies by other researchers also reported the similar observation of unequal access to iodized salt (22–24). Lack of knowledge about the link between the use of iodized salt and IDDs and the high cost of iodized salt appeared as correlates of low consumption of iodized salt in Bangladesh (22). Studies in other parts of the world have also documented the important influence of knowledge on willingness to purchase and use iodized salt (23,24).

We found almost an equal proportion of salt being adequately iodized at the households (72.9%) and retail outlets (71.7%). This might have an overall influence to equitable access to adequately-iodized salt. A recent study in Orissa reported the use of 45% and 47.7% of adequately-iodized salts at the households and retail outlets respectively (25).

Twenty-five percent of shops still sell inadequately-iodized salt. Loose salts, mostly being inadequately iodized, are sold at a cheaper cost. Ban on the production and sale of non-iodized salt also appeared to be not being effectively enforced. Moreover, the perceptions of the majority (58%) of the retail shop-owners about iodized salt and IDDs were generally incorrect. Besides other factors at the consumer level, all these factors at the retail shop level might also have contributed to less access to iodized salt in the area.

Consumption of adequately-iodized salt at the household level is substantially below the expected level of 90%. The salt-iodization programme had less access to cover those who are less aware of its importance, who are socially and economically disadvantaged, and who follow the improper practice of storage. In this context, the state IDD-control programme needs to emphasize on the improvement of the salt-iodization coverage, implement ban on the production and sale of non-iodized salt, subsidize the production and distribution of iodized salt, and focus awareness-generation campaign for the socially- and economically-disadvantaged population and shop-owners for increasing the consumption and sale of iodized salt and also for good practice of storage.

## ACKNOWLEDGEMENTS

The authors gratefully acknowledge the financial grant received from the Training in Epidemiology and Public Health Interventions Network (TEPHINET), Atlanta, GA, USA.

The authors also acknowledge the support and cooperation of the district authorities and the Department of Health and Family Welfare, North 24 Parganas, West Bengal, India. They particularly appreciate the field workers of the health system working in the study areas for their contributions to conducting the survey. The authors thank the faculty members of the Department of Community Medicine, R.G. Kar Medical College and the Department of Biochemistry, Medical College, Kolkata, West Bengal, for their support and cooperation.
